# Screening of Diabetic and Heart Failure Patients for Silent Atrial Fibrillation

**DOI:** 10.1016/j.cjco.2024.11.023

**Published:** 2024-12-04

**Authors:** Elvira Silajdzija, Ida Marie Lund, Julie Bech Jensen, Annam Pervez Sheikh, Johanne Lynge Hansen, Maya Tourkaman, Valborg Heinesen, Thomas Kallemose, Jenny Lillqvist, Clemens Steinwender, Martin Clodi, Tijn Hendrikx, Johan Engdahl, Helmut Pürerfellner, Ulrik Dixen

**Affiliations:** a Department of Cardiology, Copenhagen University Hospital, Hvidovre, Denmark; b Karolinska Institutet, Department of Clinical Sciences, Danderyd Hospital, Stockholm, Sweden; c Department of Clinical Research, Copenhagen University Hospital, Hvidovre, Denmark; d Family Medicine, Department of Public Health and Clinical Medicine, Umeå University, Umeå, Sweden; e Department of Cardiology and Medical Intensive Care Medicine, Kepler University Hospital, Linz, Austria; f Clinical Research Institute for Cardiovascular and Metabolic Diseases, Medical Faculty, Johannes Kepler University, Linz, Austria; g Department of Internal Medicine, Hospital St John of God, Linz, Austria; h Department of Medicine, Halland Hospital, Halmstad, Sweden; i Department of Cardiology, Ordensklinikum Linz Elisabethinen, Austria

## Abstract

**Background:**

Atrial fibrillation (AF) is a common heart rhythm disorder with various clinical presentations, including asymptomatic AF, known as silent AF. High-risk patients not treated with anticoagulants are at increased risk of stroke. Therefore, systematic screening has been evaluated to reduce death and cardiovascular complications. Concentrating screening efforts on high-risk populations may optimize the effectiveness of future screening strategies. The aim of our study was to determine the prevalence of silent AF in a high-risk population 65 years or older with diabetes mellitus type 2 (DM2) or congestive heart failure (CHF).

**Methods:**

A multicentre, observational, prospective cohort study of 645 patients with DM2 or CHF screened for AF in primary care and outpatient clinics in Denmark, Sweden, and Austria from 2016 to 2020. Patients were examined by intermittent electrocardiogram (ECG) recordings using a handheld ECG device 4 times daily for 2 weeks. AF was diagnosed with at least 1 recording (30 seconds) of AF. Patients with fewer than 40 recordings were excluded from the analyses.

**Results:**

Overall 3.3 %, 3.0%, and 3.9%, respectively, of the patients with DM2 and CHF, and 5.5% of patients older than 74 years were diagnosed with previously undetected AF.

**Conclusions:**

Intermittent handheld ECG screening revealed AF in 1 in every 30 patients in a high-risk population, with an increased observed risk in elderly patients.

**Clinical Registration Number:**

H-16015331.

## Résumé

**Contexte:**

La fibrillation auriculaire (FA) est un trouble du rythme cardiaque fréquent qui se manifeste de différentes façons, comme la FA asymptomatique, appelée FA silencieuse. Les patients qui présentent un risque élevé et qui ne sont pas traités par des anticoagulants courent un risque accru d’accident vasculaire cérébral. Par conséquent, le dépistage systématique a été évalué comme moyen de réduire la mortalité et les complications cardiovasculaires. Le fait de concentrer les efforts de dépistage sur les populations présentant un risque élevé peut optimiser l’efficacité de futures stratégies de dépistage. Notre étude visait donc à déterminer la prévalence de la FA silencieuse dans une population à risque élevé âgée de 65 ans ou plus et atteinte de diabète de type 2 (DT2) ou d’insuffisance cardiaque congestive (ICC).

**Méthodologie:**

Dans le cadre d’une étude de cohorte prospective observationnelle multicentrique, un dépistage de la FA a été mené chez 645 patients atteints de DT2 ou d’ICC dans des cliniques de soins primaires et de soins externes au Danemark, en Suède et en Autriche, de 2016 à 2020. Les patients ont été évalués à l’aide d’électrocardiogrammes intermittents enregistrés au moyen d’un électrocardiographe (ECG) portatif 4 fois par jour pendant 2 semaines. Un diagnostic de FA était posé lorsqu’au moins un enregistrement (30 secondes) indiquait une FA. Les patients qui comptaient moins de 40 enregistrements ont par contre été exclus des analyses.

**Résultats:**

Dans l’ensemble, 3,3 % des patients (3,0 % et 3,9 % des patients atteints respectivement de DT2 et d’ICC) et 5,5 % des patients de plus de 74 ans ont reçu un diagnostic de FA qui n’avait pas été détectée auparavant.

**Conclusions:**

Le dépistage par ECG portatif intermittent a révélé une FA chez 1 patient sur 30 dans une population à risque élevé. Le risque observé était par ailleurs accru chez les patients plus âgés.

Atrial fibrillation (AF) is a common heart rhythm disorder and a major cardiovascular challenge with a prevalence of 1.5% to 2.0% in the general population.^1^ In Europe, the prevalence is estimated to increase 2-fold in the year 2060.^2^ AF has been diagnosed in more than 3% of the population in Sweden, primarily because of improved screening of heart diseases, better health care, and longer life expectancy.^3^^,^^4^

Nonetheless, this might just be the tip of the iceberg because of underdiagnosis and, consequently, undertreatment of asymptomatic (silent) AF.^5^ Untreated, the arrhythmia is associated with an increased risk of ischemic stroke as the most severe complication.^6^, ^7^, ^8^ Silent AF is often discovered incidentally at routine examinations, preoperative assessments, population surveys, or in the wake of a stroke.^3^^,^^9^ Early detection and treatment of AF can reduce the risk of development of arrhythmia-associated heart failure, tachycardiomyopathia, and progression of cardiac remodelling of the atria and the ventricles leading to sustained AF.^10^, ^11^, ^12^ However, clear definitions of high-risk groups and which patients with a low burden of AF not needing treatment with prophylactic anticoagulation still remain unclear and are important to tailor the optimal systematic screening program.

In this study (the SILENCE study), we present the prevalence of silent AF in a high-risk population 65 years or older with 1 additional risk factor, diabetes mellitus type 2 or congestive heart failure, through intermittent electrocardiogram (ECG) recordings with a handheld device.

## Methods

### Study population

This was a multicentre, observational, and prospective cohort study. Patients were included from outpatient cardiology and diabetes clinics at Hvidovre University Hospital in Denmark, at primary care centres in Northern Sweden and from hospitals in both Linz, Austria, and Stockholm, Sweden.

**Figure 1:**
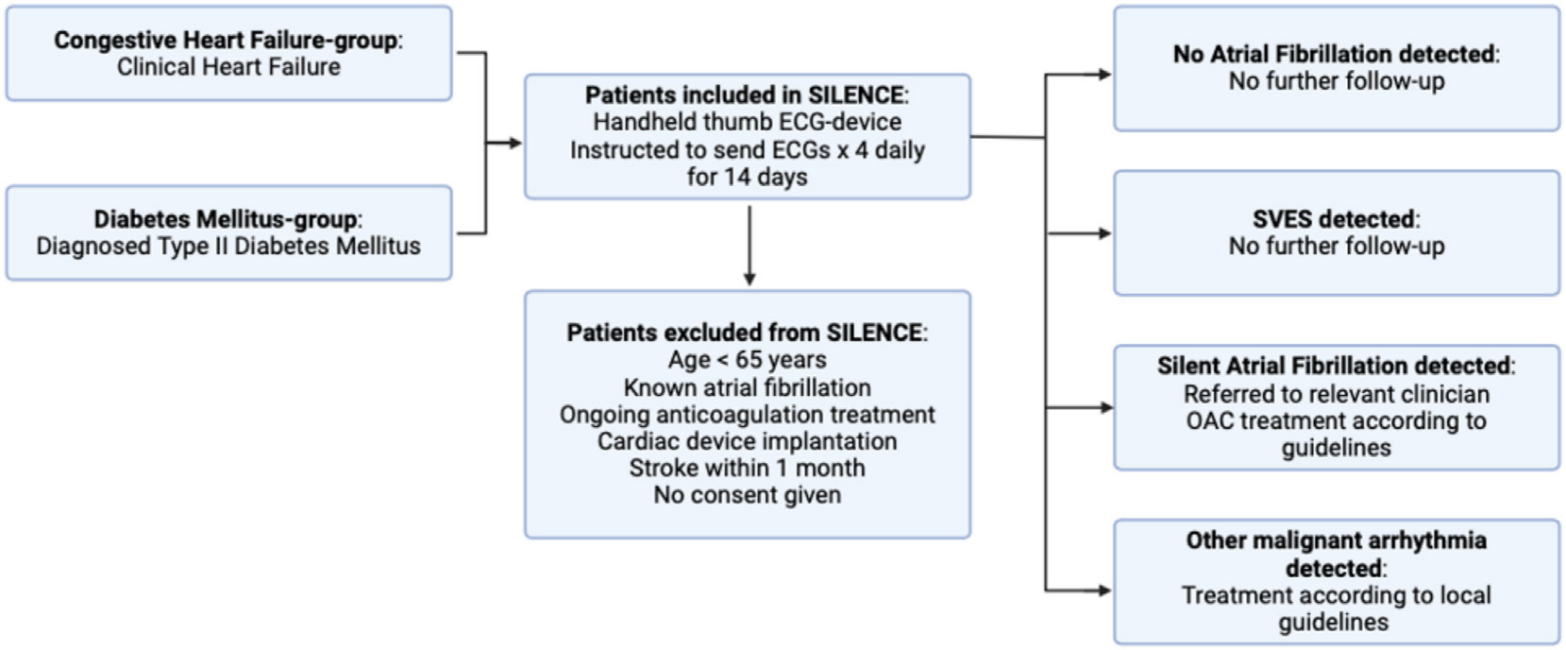
The SILENCE study. AF, atrial fibrillation; ECG, electrocardiogram; LVEF, left ventricular ejection fraction; OAC, oral anticoagulation; SVES, supraventricular extra systoles.

**Figure 2:**
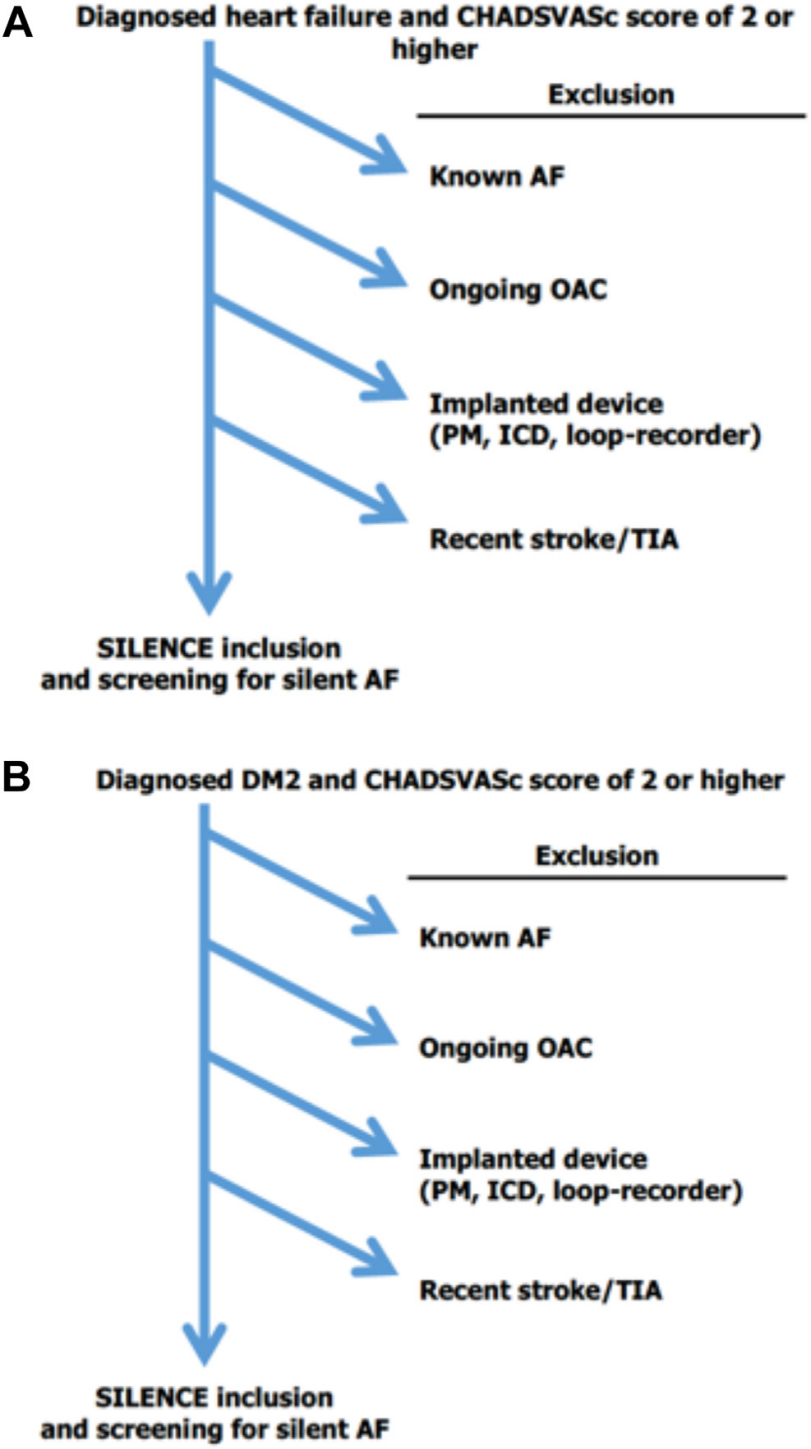
(**A**) Screening procedure: congestive heart failure. (**B**) Screening procedure: type 2 diabetes mellitus. AF, atrial fibrillation; DM2, diabetes mellitus type 2; ICD, implantable cardioverter defibrillator; PM, pacemaker; TIA, transient ischemic attack.

Individuals aged 65 years or older diagnosed with either diabetes mellitus type 2 (DM2) or congestive heart failure (CHF) were eligible for screening (Fig. 1). Thus, all study patients had CHA_2_DS_2_VASc scores of 2 or more. We defined CHF as clinical overt heart failure. At the beginning of the inclusion period, this involved a left ventricular ejection fraction (LVEF) below or equal to 40% confirmed by echocardiography. However, because of a challenging low inclusion rate, CHF was defined as clinical heart failure regardless of LVEF from 2019 onward. Patients were considered to have DM2 when meeting the clinical diagnostic criteria or in treatment for the disease and were affiliated with an experienced diabetes clinic or health care centre. Patients under the age of 65 years were not included in the study regardless of their CHA_2_DS_2_VASc score. The exclusion criteria were known AF, anticoagulation treatment with warfarin or direct oral anticoagulation (DOAC), recent stroke within a month, and cardiac device implantation (pacemaker, loop-recorder, or implantable cardioverter defibrillator) (Figure 1, Figure 2 and Figure 1, Figure 2, A and B). Patients with atrial flutter were also included.

### Sample size and screening procedure

Sample size calculations were based on an estimated prevalence of silent AF in a high-risk group. Taking earlier published results into consideration,^3^^,^^13^ the prevalence of silent AF was set at 5%. With an accepted precision of 1.5%, 811 patients should have been included.

Patients were included from of January 1, 2016 until July 1, 2020. Before giving written consent, the patients were provided with written and oral information about the study protocol. Clinical data on medical history—including history of AF, stroke/transient ischemic attack (TIA), heart failure, diabetes, vascular disease, and myocardial infarction, as well as implanted cardiac device or ongoing anticoagulation therapy—was obtained through electronic medical records.

Patients eligible for inclusion were instructed to use a handheld 2-thumb ECG device from Zenicor One (Stockholm, Sweden) 4 times daily and, in case of arrhythmia symptoms, for 14 days. Intermittent ECG recordings were efficient in diagnosing AF being 3 to 4 times more effective compared with 24-hour Holter-monitoring.^14^^,^^15^

### Detecting arrhythmia

We defined AF as an irregular supraventricular heart rhythm without P waves lasting for at least 1 full recording (30 seconds or more) or 2 separate episodes of 10 to 29 seconds. A minimum of 40 ECG measurements were needed for a patient to be compliant. A software-based algorithm excluded ECGs showing sinus rhythm. The algorithm had been validated, showing no risk of underestimating AF prevalence when allowing the algorithm to exclude up to 80% of ECG recordings.^16^ Remaining ECGs were manually analyzed by the research group according to diagnostic guidelines.

### Positive findings

Every patient with newly detected AF was referred to the local cardiology outpatient clinic, oral anticoagulation (OAC) clinic, private practitioner or primary care centre, depending on local instructions. Treatment followed European guidelines^1^^,^^17^ with respect to the patient’s age, kidney function, and previous bleeding history. Patients with inconclusive ECG findings were offered a 24-hour Holter monitoring. Patients with high-grade atrioventricular blocks or other malignant arrhythmia were handled promptly according to local guidelines (Fig. 1).

### Statistics

Descriptive statistics are presented for total AF and non-AF. Estimates are presented as median with interquartile range (IQR) for continuous variables and frequency or percentages for categorical variables.Density of the age distribution within AF and non-AF is presented graphically.

Predictive plot for probability of AF based on age was done using a logistic regression model including age as the only independent variable. Goodness of fit (GOF) test for the logistic regression model was performed by Hosmer-Lemeshow GOF test. Prevalence estimates are presented as percentage with 95% confidence intervals (CI). All analyses were done using R 4.1.0 (R Foundation for Statistical Computing, Vienna, Austria).

## Results

**Figure 3:**
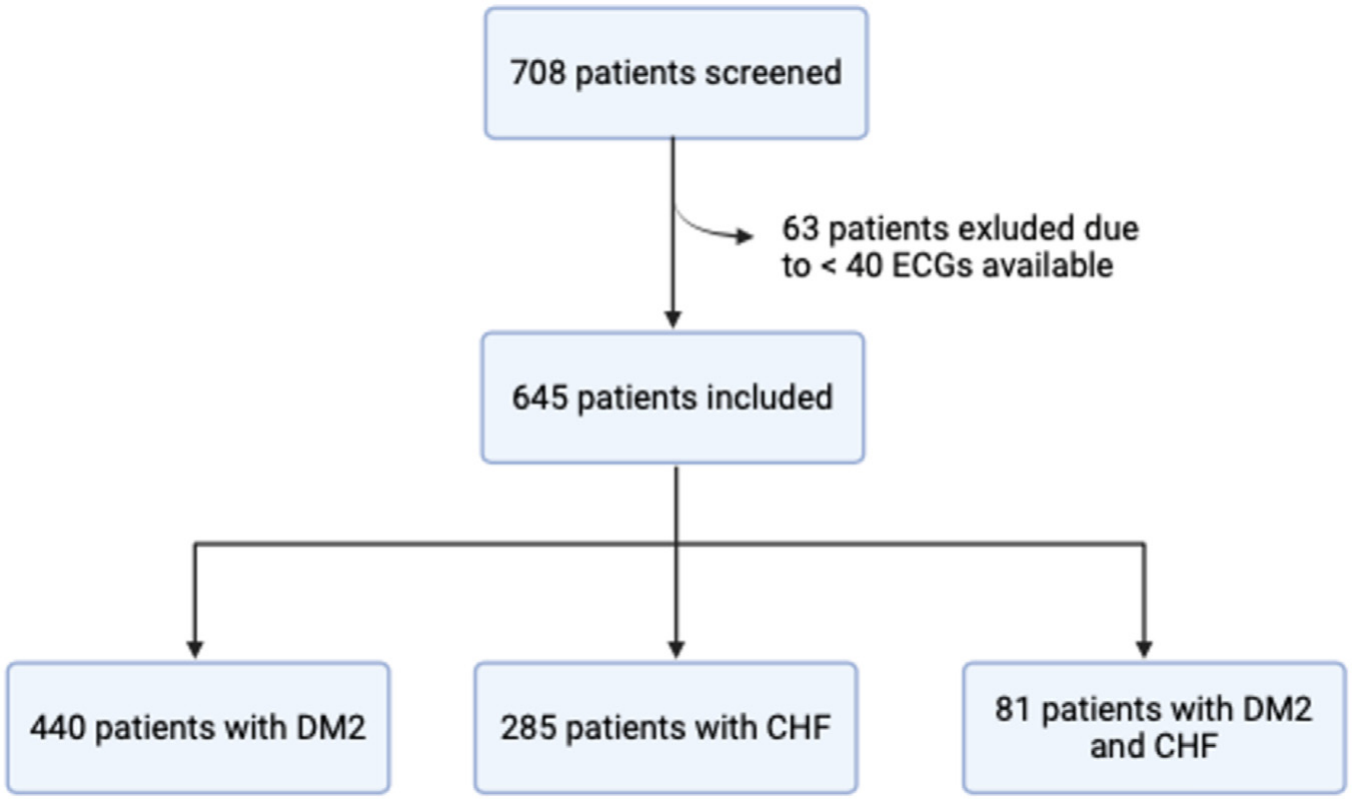
Overview of the inclusion process. CHF, congestive heart failure; DM2, diabetes mellitus type 2; ECG, electrocardiogram.

A total number of 708 patients were screened, of whom 645 were included in the analysis. Reasons for exclusion were age under 65 years or less than 40 ECGs available. Of these, 440 (68%) had DM2, 285 (44%) had CHF, and 81 (13%) had both risk factors (Fig. 3).

In total, 3.3% (CI, 2.1%-4.9%, n = 21) of the including patients were diagnosed with yet undetected AF. In the diabetes population, 3.0% (CI, 1.7%-5.0%) of the participants had AF on at least 1 ECG recording, and among the patients with heart failure, 3.9% (CI, 2.2%-6.8%) were diagnosed with AF.

The median age of the study population was 72 years (IQR: 69-77 years), and 382 participants (59.5%) were male. The median CHA_2_DS_2_VASc score was 4 (IQR: 3-5). Table 1 shows the clinical characteristics of the study population at baseline.

**Table 1:** Clinical characterstics of the study population (n = 645) at baseline

Demographics	All patients (n = 645)	Atrial fibrillation (n = 21)	No atrial fibrillation (n = 593)
Age	72 (69 : 77)	76 (74 : 79)	72 (68 : 77)
65-74	410 (63.57%)	8 (38.1%)	387 (65.26%)
75-84	209 (32.4%)	12 (57.14%)	183 (30.86%)
≥85	26 (4.03%)	1 (4.76%)	23 (3.88%)
Sex (male)	382 (59.5%)	12 (57.14%)	347 (58.81%)
BMI	28 (25 : 32)	27 (23 : 29)	28 (25 : 32)
ECGs available	57 (53 : 60)	58 (55 : 59)	57 (53 : 60)
Clinical findings			
CHF	285 (44.19%)	11 (52.38%)	264 (44.52%)
Hypertension	436 (67.6%)	13 (61.9%)	404 (68.13%)
Diabetes	440 (68.22%)	13 (61.9%)	402 (67.79%)
Stroke/TIA	61 (9.46%)	3 (14.29%)	53 (8.94%)
Vascular disease	177 (27.44%)	8 (38.1%)	163 (27.49%)
IHD	105 (16.28%)	6 (28.57%)	93 (15.68%)
IHD and PVD	12 (1.86%)	0 (0%)	12 (2.02%)
PVD	18 (2.79%)	1 (4.76%)	17 (2.87%)
Total CHA_2_DS_2_ VASc score	4 (3 : 5)	4 (3 : 5)	4 (3 : 5)

Median (interquartile range [IQR]); n (%)

BMI, body mass index; CHF, congestive heart failure; IHD, ischemic heart disease; PVD, peripheral vascular disease; TIA, transient ischemic attack.

**Figure 4:**
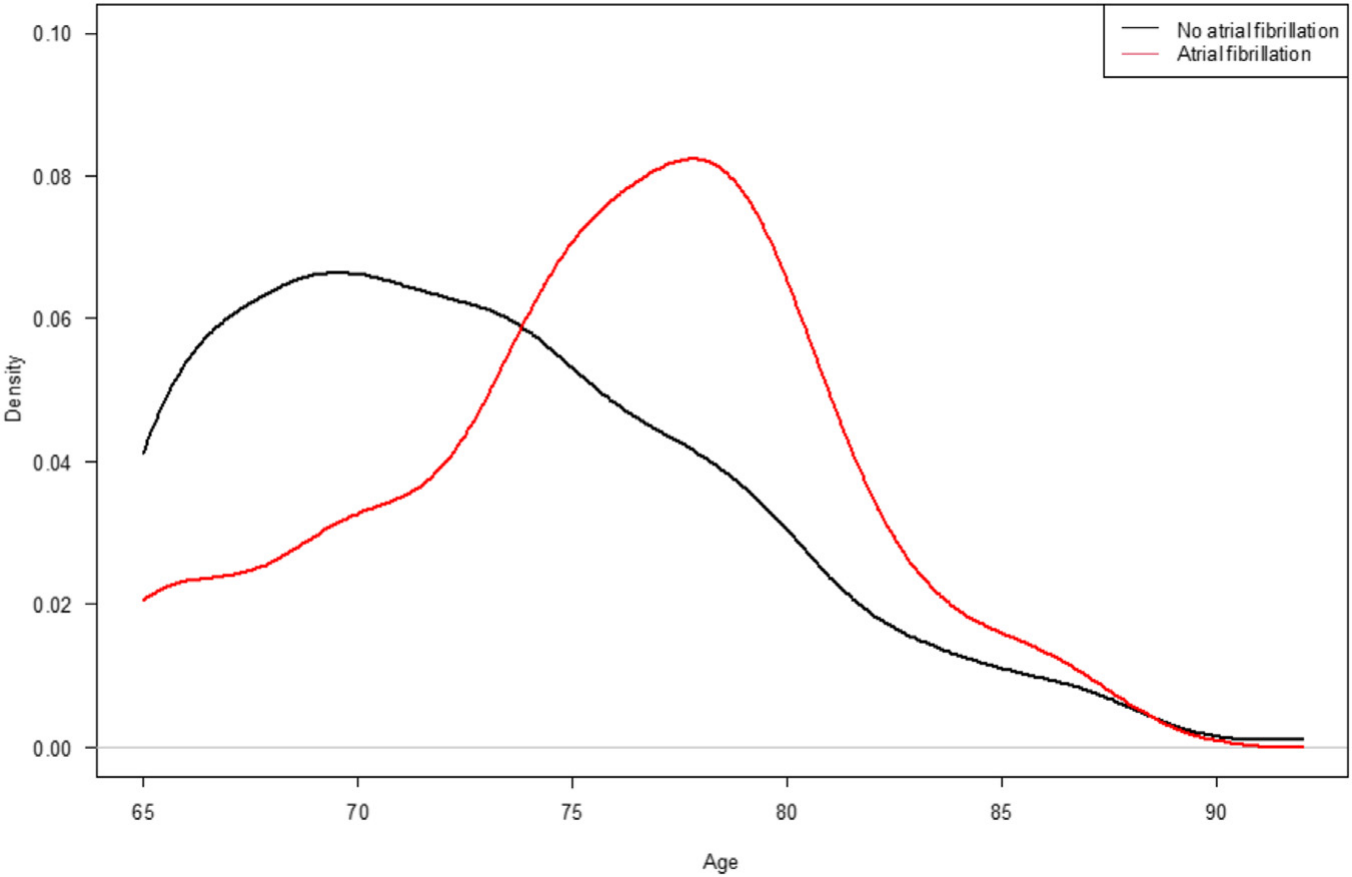
Density plot in patients with and without atrial fibrillation according to age. Density in patients with and without atrial fibrillation stratified according to age.

The median age of the participants with AF was 76 years (IQR: 74-79 years, n = 21), and 12 (57.1%) of these were male. The median CHA_2_DS_2_VASc score in the group with AF was 4 (IQR: 3-5). Thus, the AF group was significantly older compared with the screening-negative participants who had a median age of 72 years (IQR: 68-77) (Table 1, Fig. 4).

**Figure 5:**
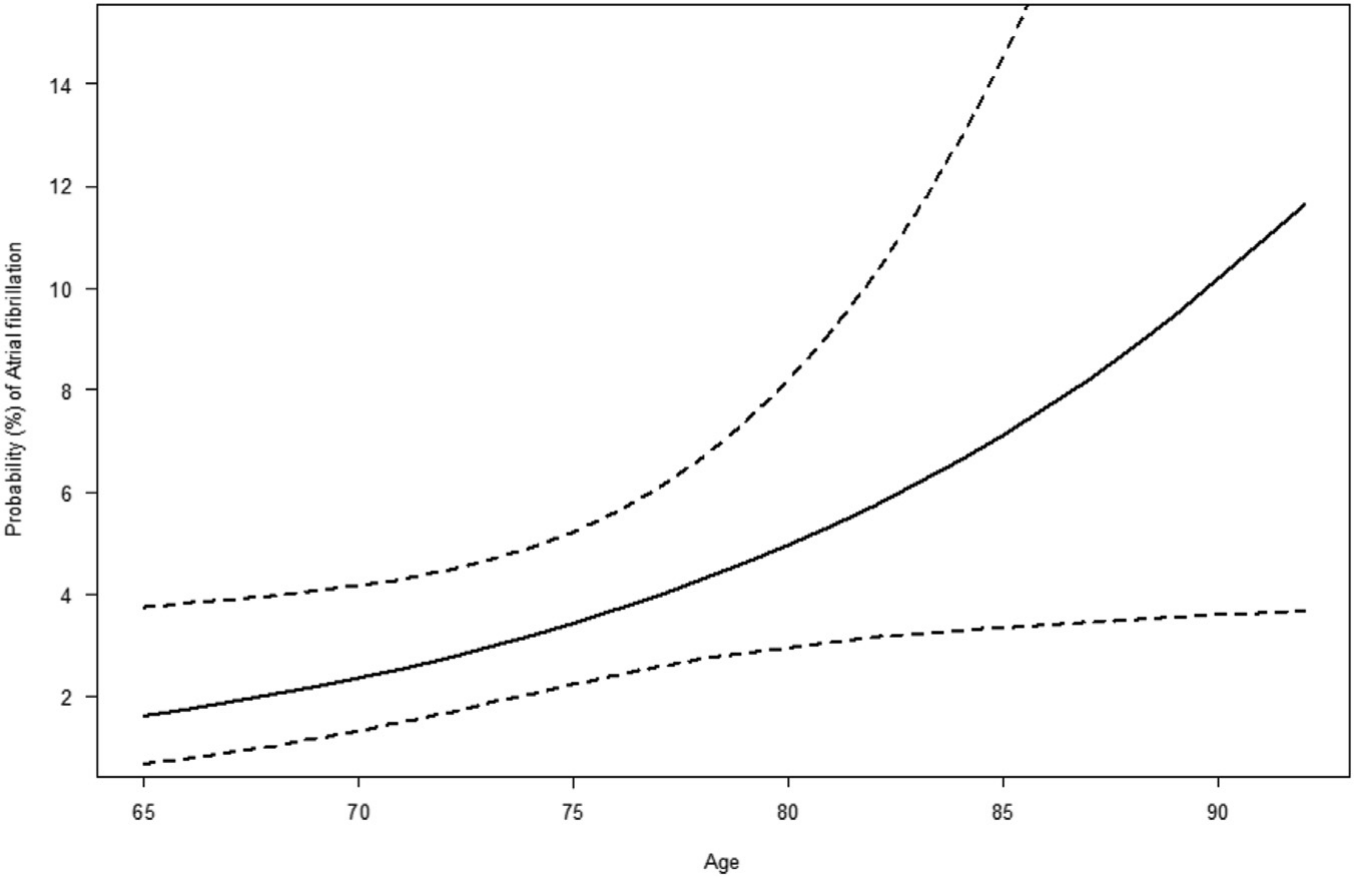
Probability of atrial fibrillation in patients stratified according to age. Change in probability (%) of atrial fibrillation according to change in age in years. **Dashed lines** indicate 95% confidence interval.

When predicting the prevalence of silent AF diagnosed according to age, the probability of AF increased with age; from 1.6 % at 65 years of age to 11.6% at age 92 years. However, uncertainty of the estimates did increase severely from approximately age 77 years (Fig. 5). Specifically, when looking at patients aged 65 to 74, the prevalence of silent AF diagnosed was 2.0% (CI, 1.0%-3.8%) and for patients 75 years or older, 5.5% (CI, 3.3%-9.2%).

## Discussion

Using a noninvasive screening method, our study identified previously undetected and untreated AF in 3.3% in 2 high-risk groups exhibiting a minimum of 2 risk factors with prevalence increasing in elderly patients.

### Importance of screening

The decision to set up systematic screening for silent AF is complex. On one hand, detection and treatment of silent AF might improve prognosis in selected patients; on the other hand, results have been contradicting when invasive screening methods and lower treatment thresholds have been used.^8^^,^^18^, ^19^, ^20^ Moreover, systematic screening requires a robust diagnostic algorithm with minimal false positive results, along with well-organized logistical settings for the follow-up of individuals identified as positive during screening. In addition, the consideration of unwarranted concerns arising from false positives or insignificant downstream findings should be integrated into the decision-making process.

For screening to be relevant, the prevalence of silent AF must advocate for screening and the procedure must be cost effective. According to a cost-effectiveness analysis, screening resulted in additional patient-years with detected AF, fewer strokes and gained quality-adjusted life years (QALYs) per individual.^21^

Current guidelines^1^ recommend opportunistic screening for silent AF; however, with a class 2A evidence level, systematic screening of people aged 75 to 76 years can be supported.

Silent AF has been shown to convey an increased risk of stroke or TIA in high-risk populations.^22^, ^23^, ^24^ Complications of stroke can be fatal^25^ and potentially devastating for patients and their relatives with risks of aphasia, paralysis, cognitive limitations, and depression.^25^, ^26^, ^27^ The importance of screening for AF is supported by a study by Svennberg et al.,^28^ in which the researchers found a reduction of a composite endpoint, including stroke, in patients offered anticoagulant medication after AF was detected with screening.

The importance of our findings is supported by recent studies. Healey et al.^29^ recently found that among patients with subclinical AF (n = 4012), apixaban resulted in a lower risk of stroke or systemic embolism than aspirin, indicating the significant benefit in treating patients with silent or subclinical AF and thus calling for early detection and treatment of these patients in order to reduce morbidity and mortality.

Similarly, Lopes et al.^30^ found in the Apixaban for the Reduction of Thrombo**e**mbolism in Patients With Device-Detected Subclinical Atrial Fibrillation (ARTESiA) study that in subclinical AF with CHA_2_DS2-VASc ≥ 4, the benefits of OAC outweigh bleeding risk when comparing apixaban with aspirin. This underlies the importance in identifying patients with AF subclinically by screening. Furthermore, the Early Treatment of Atrial Fibrillation for Stroke Prevention (EAST-AFNET) study^10^ demonstrated that early and consequent heart rhythm control led to reduced risk of death and cardiovascular complications, regardless of symptoms.

This is especially of importance, considering that device-detected subclinical AF carries a higher stroke risk than no atrial tachyarrhythmias, termed high-rate episodes (AHREs), and that it often progresses to clinical stage with clinical AF having a higher risk of stroke. Detecting AF in the early stages can thus delay this progression and reduce the risk of potentially fatal stroke complications. This is especially relevant, given that AHREs are present in approximately one-third of device carriers.^31^

However, current results from large-scaled studies of systematic screening in high-risk groups have been conflicting. The Swedish randomized Systemic Screening for Atrial Fibrillation (STROKESTOP) study^3^ extended invitations to more than 13,000 residents aged 75 to 76 years from 2 regions in Sweden. The individuals were invited to participate in intermittent screening for AF using a handheld ECG device. The study outcomes were compared with those of a matched control group who did not receive invitations to participate in the study. Participants diagnosed with AF were offered anticoagulation as stroke prophylaxis. Systematic intermittent 2-week screening was associated with a small but significant reduction in the risk of death or cardiovascular complications.

The study, nonetheless, did not specify which patient groups within the age of 75 to 76 years should be screened for AF to have a significant reduction in cardiovascular complications, including stroke. With health care systems internationally calling for resource allocations and priority setting,^32^ this is warranted. In our study, we specify which patient groups are at increased risk for silent AF—and thus stroke—and therefore would particularly benefit from screening, adding to our current knowledge from the STROKESTOP study.

Another large-scale study of systematic screening is the Danish Implantable **Loop** Recorder Detection of Atrial Fibrillation to Prevent Stroke (LOOP) study,^18^ which used an invasive screening method with an implantable loop recorder in a high-risk group with a mean age of almost 75 years. Despite diagnosing nearly 4 times more patients in the screening group compared with the control group and providing anticoagulant treatment upon detecting at least 6 minutes of AF, the study revealed no significant improvement in prognosis concerning mortality or cardiovascular complications, including stroke or systemic embolism.^18^

Therefore, it appears to be crucial to pinpoint and screen actual high-risk patients based on the results of the LOOP study^18^ and not to diagnose and treat patients with a very low burden of AF found from invasive and continuous screening methods. Our study suggests that systematic screening for silent AF seems reasonable in individuals 65 years or older with DM2 or CHF.

However, screening with devices can be time consuming and may result in incidental findings that physicians must assess, potentially demanding additional resources. The growing use of consumer devices and wearables, such as smartwatches, complicates this issue with physicians having to evaluate ongoing cases of silent atrial fibrillation.^33^ This highlights the need for clearly defined risk groups for screening. However, in a real-world setting using a handheld ECG device potentially alleviates time for both the physician and patient. Nevertheless, an integration between consumer-driven technology and systematic screening approaches could ultimately refine AF management strategies and broaden their reach. Moreover, further research is needed in regards to determination of screening intervals in order to detect the most actionable episodes of atrial fibrillation.

### Limitations

In our study, we aimed to report prevalence of silent AF in 2 high-risk groups. Because of halted inclusion in the COVID-19–driven lockdown, we were unable to include the number of patients calculated in our sample size estimation. This is an important limitation that should be taken into consideration when interpreting the SILENCE study results.

It is also important to note that our cohort consisted of patients from both primary care and outpatient clinics, potentially including more patients with more morbidities from the outpatient clinics compared with those from primary care clinics. This potentially makes our results challenging to generalize to patients from primary care clinics.

Furthermore, intermittent recordings could underdiagnose paroxysmal AF, as the patient could be in sinus rhythm when the recordings are made. Regarding paroxysmal AF, 24/7 ECG monitoring thus might appear more suitable.

Nevertheless, we report data from more than 600 high-risk patients with consistent observations of 3% to 5.5% undiagnosed AF in patients with DM2 and CHF. Compared with other setups for systematic screening, the prevalence of undetected AF is slightly higher in our study.^3^^,^^34^

Thus, our results can merely be regarded as pieces in the complex process toward providing public systematic screening or a decision to refrain from more than opportunistic screening. The large-scaled AF-screening **S**creening for **A**trial **F**ibrillation With **E**CG to **R**educe Stroke (SAFER) study^35^ in Europe and Australia could potentially deliver a conclusion with regard to the prognostic effect of systematic screening for silent AF. Our study provides new knowledge of prevalence of silent AF diagnosed with intermittent noninvasive ECG measurements in elderly patients with heart failure or diabetes mellitus type 2 to qualify future decision making on possible screening programs.

## Conclusions

In elderly patients with heart failure or DM2, screening for silent AF revealed silent AF in 1 of every 30 patients and in approximately 1 of every 20 patients older than 74 years of age.
